# Cost–related unmet need for healthcare services in Kenya

**DOI:** 10.1186/s12913-020-05189-3

**Published:** 2020-04-17

**Authors:** Purity Njagi, Jelena Arsenijevic, Wim Groot

**Affiliations:** 1 United Nations University-MERIT, Maastricht Graduate School of Governance, Maastricht University, Maastricht, The Netherlands; 2 Utrecht University School of Governance, Faculty of Law, Economics and Governance, Utrecht University, Utrecht, the Netherlands; 3 Department of Health Services Research, Faculty of Health Medicine and Life Sciences, Maastricht University, Maastricht, The Netherlands

**Keywords:** Cost-related, Unmet need, Multilevel analysis, Healthcare, Equity

## Abstract

**Background:**

The assessment of unmet need is one way to gauge inequities in access to healthcare services. While there are multiple reasons for unmet need, financial barriers are a major reason particularly in low- and middle-income countries where healthcare systems do not offer financial protection. Moreover, accessibility and affordability are paramount in achieving universal health coverage. This study examines the extent of unmet need in Kenya due to financial barriers, the associated determinants, and the influence of regional variations.

**Methods:**

We use data from the 2013 Kenya household health expenditure and utilization (KHHEUS) cross sectional survey. Self-reported unmet need due to lack of money and high costs of care is used to compute the outcome of interest. A multilevel regression model is employed to assess the determinants of cost-related unmet need, confounding for the effect of variations at the regional level.

**Results:**

Cost-related barriers are the main cause of unmet need for outpatient and inpatient services, with wide variations across the counties. A positive association between county poverty rates and cost-related unmet is noted. Results reveal a higher intraclass correlation coefficient (ICC) of 0.359(35.9%) for inpatient services relative to 0.091(9.1%) for outpatient services. Overall, differences between counties accounted for 9.4% (ICC ~ 0.094) of the total variance in cost-related unmet need. Factors that positively influence cost-related unmet need include older household heads, inpatient services, and urban residence. Education of household head, good self-rated health, larger household size, insured households, and higher wealth quintiles are negatively associated with cost-related unmet need.

**Conclusion:**

The findings underscore the important role of cost in enabling access to healthcare services. The county level is seen to have a significant influence on cost-related unmet need. The variations noted in cost-related unmet need across the counties signify the existence of wide disparities within and between counties. Scaling up of health financing mechanisms would fundamentally require a multi-layered approach with a focus on the relatively poor counties to address the variations in access. Further segmentation of the population for better targeting of health financing policies is paramount, to address equity in access for the most vulnerable and marginalized populations.

## Introduction

Delaying or forgoing treatment may have negative effects on health status among different population groups [1]. Furthermore, forgoing necessary treatment might impose a financial burden in the long run, or even lead to poorer health. At least half of the world’s 7.3 billion population are reported to not receive the essential health services they need [2]. This is more profound in developing countries where many people go without health care from which they could benefit greatly [3]. The reasons for forgoing healthcare can be found in the numerous barriers that hinder access such as [4] financial, organizational, social, psychological or cultural barriers. These barriers may limit use of healthcare services and impose large healthcare coverage gaps among different population groups [5]. One method of gauging equity of access to services is through assessing unmet needs for health care [6, 7]. Unmet need is implicitly defined as the difference between the services judged necessary and the services actually received, resulting from barriers related to accessibility, availability and acceptability [8]. There are two recommended approaches for measuring unmet need. On the one hand, one can use a ‘clinical’ assessment based on relevant clinical guidelines. On the other hand, there is a ‘subjective’ measure based on the individual’s own assessment not to have received healthcare when needed because of access barriers beyond their control [9]. The subjective measure is a widely used approach due to its feasibility as many surveys include self-reports by individuals on the time, they needed care but did not receive it [10, 11].

Poor functioning health financing systems and the cost of health services may deter people from seeking healthcare when they need it in order to avoid financial burden. This is especially observed when direct out of pocket (OOP) payment are involved [12, 13]. Financial access is thus critical, given that it can lead to catastrophic costs and/or impoverishment [14, 15]. When there are no direct costs at the time of access to healthcare services (out of pocket patient payments (OOP) for example), then catastrophic health expenditure can be prevented [16]. Many studies have used catastrophic health expenditure to gauge the extent of financial protection. However, given CHE occurs in the form of direct and indirect health expenditure it is only incurred if sick individuals actually seek the needed healthcare. This means that the small incidence of CHE in some countries may create the impression of a greater degree of financial protection than what it provided by the system [15]. Furthermore, the analysis of CHE may suffer from selection bias. For instance a low incidence of catastrophic spending might simply reflect a situation in which only a few people get the health care they need [17], and we do not observe people who need care but are unable to get it.

In case of the Kenya health care system, inequities in accessing the healthcare services can be described as problems in regional discrepancies in the health service distribution, disparities in resource allocations, and inequitable access to quality health services [18, 19]. Significant regional inequities remain where northern counties, rural households and ethnic minorities are reported to have worse healthcare coverage [20]. Recent analysis show regional disparities in access, in that some regions are less likely to go without medical care, while other regions experience difficulty in obtaining medical care [21, 22].

The inequities in accessing healthcare services are also related to unmet need. A large variation in unmet healthcare needs is reported across counties ranging between 4.1 to 40.4%, with the cost being one of the top reasons why people forgo care [23]. Furthermore the counties vary by socioeconomic composition, as some are pre-dominantly rural while others are urban [24]. Additionally, the numbers of those who do not seek care in Kenya due to costs is particularly high in the rural areas [25].

Studies on unmet need for care in Kenya and the region have primarily examined overall reasons for unmet need for specific health services, particularly reproductive services [26–30]. Others have focused on barriers to utilization of services such as non-availability of drugs, staffing inadequacy among others [31, 32]. To our knowledge, there has been no study that has looked at the factors influencing unmet need for healthcare due to costs alone. Analysis of the reasons for unmet need can be important in focusing policy actions [9]. This study adds to the existing literature on financial access by first, examining the extent of cost-related unmet need for healthcare and second, explaining how much of the cost-related unmet need is attributable to the differences in individual characteristics, and the effect of the variations at the regional (county) level. Geographical assessment of unmet need is critical in the Kenyan context given the recent (2013) decentralisation of the health care system to new sub-national governance units (counties). Moreover, the counties have the responsibility for healthcare financing and provision of services. This study applies a multi-level regression analysis model to provide for factors that are contextual at the individual/household level, adjusting for variations at the county level. Furthermore, many empirical studies are reported to focus on individual level and/or aggregate level inequities and not paying attention to multilevel structures [33]. A multi-level analysis provides a more nuanced understanding of the drivers that are influential at the different levels. Disaggregating unmet need discerns those reasons that are relevant to policymakers, and those reflecting individuals’ and households’ preferences and taste [34]. The findings of this study are relevant in informing the healthcare financial reforms in Kenya both at the national and at the county level, to better improve access to services to underserved populations, and hence accelerate achievement of universal health coverage (UHC) across all regions.

## Methods

### Data

This study uses data from the Kenya household health expenditure survey (KHHES) implemented by the Ministry of Health in collaboration with Kenya National bureau of statistic every 5 years. This is a national representative household survey aimed at collecting data on household health care utilization. The survey collects data on individuals who were ill, those who sought care, and the reasons for not seeking care when ill. Other data collected include demographic and socioeconomic characteristics of the individuals and households. This study utilizes data from the 2013 survey which covered Kenya’s 44 out of the 47 counties. A total of 33,675 households (152,566 individuals) were interviewed from 1347 selected clusters; 814 (60%) were rural and 533 (40%) urban clusters with a response rate of 87.7%.

### Variables

#### Outcome variable

Unmet need for healthcare is seen to cover a spectrum of healthcare needs that are not optimally met. On one hand is “unexpressed demand” referring to people with healthcare needs who are not aware of them, or who do not to seek healthcare. On the other hand is “expressed demand” referring to healthcare needs that are sub-optimally met [35].

This study uses a dependent (outcome) variable related to “unexpressed demand”, which is the subjective perception of not receiving appropriate care when needed [36]. We look at whether people with healthcare needs choose not to seek care due to the high cost of care and lack of money, referred to as self-reported ‘cost-related unmet need’ for healthcare services. We construct the cost-related unmet need for healthcare variable from two sets of questions:

(i) Outpatient - Was <NAME> ill in the last 4 weeks? If Yes, …… did <name> visit/consult a health provider?
(ii) Inpatient - Did [Name] need to be admitted in a hospital in the last 12 months? If Yes ……, was <name> admitted?

The reasons why healthcare was not sought when needed are derived from two closed ended questions framed as follows; Outpatient - “If No ……, what was <name>‘s main reasons for not seeking care?” and Inpatient “If No ……., why was <name> not admitted?”. The responses are a pre-defined set of multiple-choice categories allowing for multiple responses. They include; lacked money, prescribed drugs were not available, self-medication, poor quality of service, high cost of care, religious/cultural issues, fear of discovering serious illness, long distance to the provider and illness not considered serious enough.

#### Explanatory variables

Based on Andersen’s Health Behaviour Model we distinguish three groups of factors that affect access to healthcare services: need, predisposing and enabling factors [37, 38]. We thus classify the independent variables into (i) predisposing factors such as: age of household head, gender of household head, education level of household head and employment status of household head, (ii) need factors such as: type of service sought (inpatient/outpatient care), self-rated health status and chronic illness, and (iii) enabling factors such as: household wealth, household size, insurance status, and residence (rural/urban).

### Analysis

**Fig. 1:**
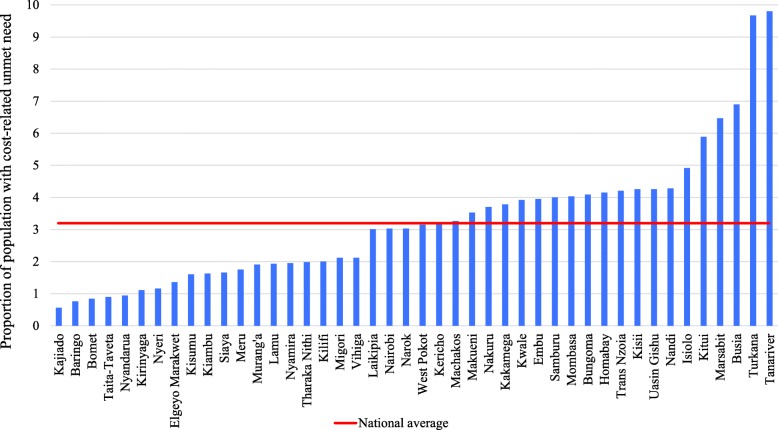
Cost-related unmet need for healthcare by counties

**Fig. 2:**
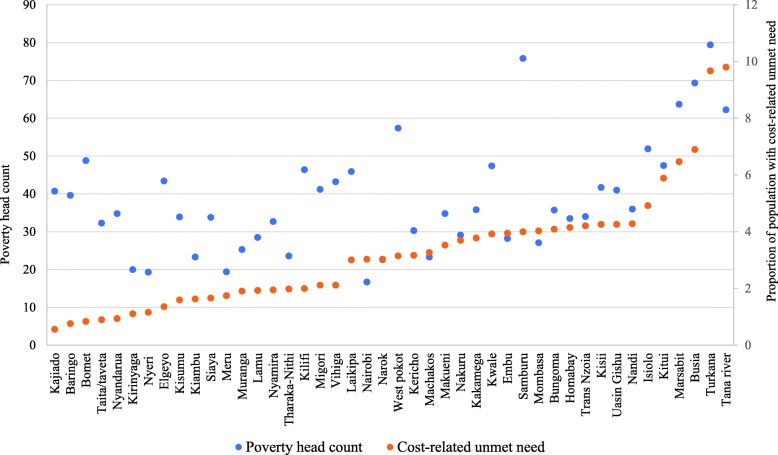
Cost-related unmet need for healthcare by county poverty rates. Poverty rates data from the Kenya Integrated Household Budget Survey(KIHBS) 2015/16 report

This study applies a multilevel logistic regression model to analyse the association and variability between cost-related unmet need for care, and the independent factors. Multilevel analysis is preferred given that it allows for the simultaneous examination of the effects of group-level and individual-level factors and groups are perceived as related, that is coming from a larger population of groups [39]. Additionally, multilevel analysis accounts for dependencies of observations within groups [40]. Multilevel (logistic) regression disentangles the within-cluster effects from the between-cluster effects [41]. Relative to conventional models, multilevel models provide a more accurate and comprehensive description of relationships in clustered data [42]. In this study, individuals and households are nested within regions known as counties, thus we use counties as the group level variable. First, we present the proportion of people who face unmet need and the different reasons for unmet needs (see Table 1), then we present descriptive statistics for unmet need related to costs (see Fig. 1 and Fig. 2), and the results related to logistic multilevel regression (see Table 3). We fit a Pearson’s correlation coefficient test statistic to assess the association between poverty rates, and cost-related unmet need for healthcare services. A log likelihood ratio test is used to assess the goodness of fit between the general logistic model and the multilevel model. Additionally, this study applies two commonly used information criteria - Alkaike’s information Criterion(AIC) and the Bayesian Information Criterion (BIC), to assess the relative model goodness of fit [43].

**Table 1:** Unmet need for healthcare services and the reasons for unmet need

Variables	Outpatient		Inpatient		Total	
	N	%	N	%	N	%
Was ill and Needed admission	36,901	88.3	4882	11.7	41,783	
Did not seek healthcare services	3009	86.9	453	13.1	3462	
Percentage with unmet need		**8.2**		**9.3**		**8.3**
Percentage with cost-related unmet need		**2.9**		**5.2**		**3.2**
**Reasons for unmet need**						
Cost-related (Lack of money and high costs)	1081	35.9	256	56.5	1337	38.6
Illness was not considered serious	1070	35.6	88	19.4	1158	33.5
Self-Medication	646	21.5	32	7.1	678	19.6
Long distance to the health provider	49	1.6	8	1.8	57	1.7
Religious/cultural reasons	36	1.2	5	1.1	41	1.2
Fear of serious illness	11	0.4	1	0.2	12	0.4
Perceived poor quality of service	8	0.3	4	0.9	12	0.4
Perceived lack of drugs at the facilities	10	0.3	4	0.9	14	0.4
Other reasons (Not specified)	98	3.3	55	12.1	153	4.4
**Total (N)**	**3009**		**453**		**3462**

## Results

### Proportion of population with unmet need for healthcare services

The analyses show that out of those who needed health care services 88.3% needed outpatient services within the last 4 weeks, whereas 11.7% needed inpatient services within the last 12 months. However, 8.3% did not seek the needed healthcare services. Unmet need for healthcare services was higher among those that needed inpatient services at 9.3%, relative to those that needed outpatient services at 8.2%. Table 1 shows the proportion with unmet need, and the various reasons for unmet need for healthcare services. The top reasons why people had to forgo care were cost-related (38.6%), Illness not considered serious (33.5%), and self-medication (19.6%). Cost-related reason was higher for inpatient services (56.5%) compared to outpatient services (35.5%).

Of those who needed outpatient or inpatient services, 3.2% did not seek the needed healthcare services due to cost, that is high cost of the services and/or lacked the money. Our analysis show that cost-related unmet need is higher for inpatient services at 5.2% compared to the outpatient services at 2.9%. Additional file 1 further presents the distribution across counties of those that did not seek healthcare services due to cost-related reasons, and other reasons including no drugs, self-medication, poor quality service, religious/cultural reasons, fear that the illness is serious, distance to provider and illness not considered serious.

Figure 1 shows the distribution of those who experienced cost-related unmet need by counties. The analysis shows a wide variation in cost-related unmet need among the counties from < 1 to 9.8%. Nearly half (43%) of the counties had cost-related unmet need higher than the national average of 3.2%. We observed that several counties with a prevalence of unmet need of < 1%, had small samples (N) of up to < 10 persons with cost-related unmet need (See Additional file 1). In light of this, we run a sensitivity analysis on the multilevel model to assess the robustness of the findings without the 9 counties that have a cost-related unmet need of less than 10%.

### Descriptive analysis of the population with cost-related unmet need for healthcare

The distribution of those who experienced cost-related unmet need for healthcare by the socio-economic characteristics is presented in Additional file 2. Households head above 40 years accounted for the majority of those who had cost-related unmet need (64.3%). The majority (81.1%) of those who experienced cost-related unmet need came from households with a head with primary education only. Persons with cost-related unmet need from male headed households were 68.1%, similarly 82.5% with cost-related unmet need had an employed (formally and informally) household head. More than half (56.3%) of those with cost-related unmet need self-rated their health as good. Only 26.2% of those with cost-related unmet need had chronic illness. Cost related unmet need for care was experienced by 42.3 and 31.7% of the people from medium and large size (7+) households respectively. Only 7.3% of those who experience cost related unmet need for care were from insured households. Rural households accounted for majority (70.3%) of the individuals who experienced cost-related unmet need for healthcare. Of the individuals who experienced cost-related unmet need for care, the majority were from poorest households (38.1%), while the least were from among the richest households (4.8%).

There was a statistically significant positive correlation (Pearson’s r 0.638; *p*-value < 0.0001) between the country poverty rates, and cost-related unmet need for healthcare. The majority of the counties that experienced high cost-related unmet need for healthcare had high poverty rates as shown in Fig. 2.

### Multilevel analysis of cost-related unmet need for healthcare

We fit both the general logistic regression model, and the multilevel regression model (see Additional file 3). Although the estimates from both models show small differences, the log likelihood test confirms that there is a statistically significant difference between the two models (chi^2^ = 257.14, *P*-value < 0.001). Relative to the logistic regression model, the multilevel model has the lowest AIC and BIC, indicating it is the better fitting model as illustrated in Table 2. This demonstrates that controlling for the county level variation leads to a significant improvement of the model, relative to running a general regression model. We therefore discuss the results from the multilevel model which controls for between counties’ variation.

**Table 2:** AIC and BIC for the logistic and multilevel model

	Obs	ll (null)	ll (model)	df	AIC	BIC
Logistic Model	41,646	− 5889.088	− 5554.914	20	11,149.83	11,322.57
Multilevel Model	41,646	.	− 5426.346	21	10,894.69	11,076.07

Table 3 presents the analysis from the multilevel logistic regression models. We fit three models including an outpatient model 1, an inpatient model 2, and an overall model 3 controlling for outpatient and inpatient services. The results show the association of the predisposing, need and enabling factors on cost-related unmet need for healthcare services at the individual level confounding for the county level. Overall, the results show an estimated intraclass correlation coefficient (ICC) of 0.094, meaning that 9.4% of the variance in cost-related unmet need is attributable to county level variations. This is close to the 0.091 ICC reported in the outpatient model, indicating the county level accounted for 9.1% of the variation in outpatient cost-related unmet need. Equally, we note a higher ICC of 0.359 in the inpatient model, suggesting that county level variation accounts for 35.9% of the inpatient cost-related unmet need. Given that 9 counties had a very low sample (< 10) with cost-related unmet need, a separate multilevel model is fitted without these counties to assess the robustness of the findings. The intraclass correlation coefficient(ICC) is 6% relative to 9.4% in the multilevel model with all the counties. Additionally, the direction of the effect of the explanatory factors on cost-related unmet need is the same in both models. Furthermore, the confidence intervals for all the factors are overlapping meaning there is no significant difference between the two models. Finally, we run a log-likelihood ratio test which indicates no statistically significant difference between the two models.

**Table 3:** Multilevel regression models for cost-related unmet need for healthcare

Factors	Categories	Model 1: Outpatient		Model 2: Inpatient		Model 3: Overall	
		OR	[95% CI]	OR	[95% CI]	OR	[95% CI]
**Predisposing factors**							
*Gender*	Male [RC]						
	Female	1.015	(0.88–1.17)	1.115	(0.82–1.52)	1.019	(0.89–1.16)
*Age group HH*	Below 25 years [RC]						
	25–40 years	1.260	(0.85–1.87)	1.452	(0.62–3.39)	1.332	(0.94–1.89)
	40+ years	1.926***	(1.31–2.84)	1.175	(0.50–2.76)	1.793**	(1.26–2.55)
*Education level of HH head*	None [RC]						
	Primary	1.057	(0.66–1.69)	0.364	(0.11–1.21)	0.956	(0.62–1.46)
	Secondary	0.772	(0.47–1.27)	0.229**	(0.06–0.79)	0.669	(0.43–1.05)
	Tertiary	0.488**	(0.26–0.91)	0.340	(0.08–1.31)	0.534*	(0.31–0.92)
*Employment status*	Not employed [RC]						
	Employed	1.009	(0.83–1.22)	0.668**	(0.45–0.98)	0.931	(0.79–1.10)
**Need Factors**							
*Type of service*	Outpatient [RC]						
	Inpatient	NA		NA		1.950***	(1.68–2.26)
*Self-rated health*	Poor [RC]						
	Satisfactory	1.108	(0.89–1.37)	0.613**	(0.41–0.92)	0.942	(0.79–1.13)
	Good	0.661***	(0.54–0.81)	0.348***	(0.24–0.51)	0.567***	(0.48–0.67)
*Chronic Illness*	No Chronic Illness [RC]						
	Chronic illness	1.163	(0.99–1.37)	0.822	(0.59–1.15)	1.067	(0.92–1.23)
**Enabling factors**							
*Insurance status*	Not insured [RC]						
	Insured	0.562***	(0.43–0.74)	0.346***	(0.22–0.55)	0.505***	(0.40–0.64)
*Household size*	1–3 Small [RC]						
	4–6 Medium	0.698***	(0.59–0.82)	0.677**	(0.46–0.99)	0.693***	(0.60–0.80)
	7+ Large	0.647***	(0.54–0.77)	1.192	(0.80–1.77)	0.717***	(0.61–0.84)
*Residence*	Rural [RC]						
	Urban	1.280**	(1.10–1.49)	1.358	(0.95–1.94)	1.259**	(1.09–1.45)
*Wealth Index*	Poorest [RC]						
	Second	0.826*	(0.69–0.98)	0.921	(0.63–1.30)	0.845*	(0.72–0.99)
	Middle	0.643***	(0.53–0.78)	0.558**	(0.36–0.86)	0.632***	(0.53–0.75)
	Fourth	0.438***	(0.34–0.56)	0.532**	(0.33–0.85)	0.470***	(0.38–0.58)
	Richest	0.371***	(0.26–0.53)	0.131***	(0.05–0.32)	0.314***	(0.23–0.44)
	_cons	0.040***	(0.02–0.08)	0.372	(0.08–1.75)	0.055***	(0.03–0.10)
*Random effects*	/lnsig2u [County variance]	−1.113	(−1.62--0.61)	0.613	(−0.01–1.24)	−1.074	(−1.57- –0.57)
	sigma_u [Residual variance]	0.573	(0.45–0.74)	1.359	(0.99–1.85)	0.585	(0.45–0.75)
	Rho [Intraclass correlation (ICC)]	0.091	(0.06–0.14)	0.359	(0.23–0.51)	0.094	(0.06–0.15)
	Log likelihood		− 4513.35		− 818.16		−5426.35
	Wald chi2(18)		354.35		129.25		443.84
	No. of observations		36,783		4863		41,646
	No. of groups		44		44		44

P < 0.05*, 0.01** and 0.001***; RC Reference Category, HH Household, NA Not Applicable, OR Odds Ratio

The analysis shows a positive association between cost-related unmet need for health care and older household heads (+ 40 years) in both the outpatient and overall model. Relative to households with younger heads (below 25 years), older households have 79 and 92% higher odds of unmet need for healthcare due to cost in the overall and outpatient model respectively. In addition, household heads with tertiary education have 47 and 52% lower odds of cost-related unmet need for healthcare relative to those with no education in the overall and outpatient model respectively. Although in the inpatient model being an employed households’ head was associated with 33% lower odds of experiencing cost-related unmet need, in the outpatient and overall model, employment status of the household head had no significant association with cost-related unmet need.

The overall model shows seeking inpatient services was associated with 95% increase in the odds of cost-related unmet need. There was a negative association between good self-rated health and cost-related unmet need across the three models, with 44% reduced odds of unmet need for care due to costs in the overall model relative to poor self-rated health. Insured households were 50% less likely to have cost-related unmet relative to uninsured households, meaning the odds were even in the overall model. Household size was negatively associated with cost-related unmet need for care across the three models. For instance, medium and larger households had 31 and 29% lower odds of cost-related unmet need respectively, relative to those from small households.

Urban residents were 25%in the overall model and 28% in the outpatient model, more likely to have cost-related unmet need for care relative to their rural counterparts. However, there was no association between cost-related unmet need and residence in the inpatient model. In all the three models, wealth status of the household was negatively associated with the cost-related unmet need for healthcare with the likelihood reducing as you move up the wealth quintiles. In the overall model, households in the richest quintile were 69% less likely to have unmet needs, while for the, fourth quintile this was 53%, the middle quintile had a 37%, and second quintile had 15% less chance to have cost-related unmet need for healthcare relative to the those in the poorest quintile.

## Discussion

The findings show that 8.3% of those who needed outpatient care or inpatient care did not seek healthcare services with inpatient unmet need being higher relative to outpatient unmet need. This is lower than the percentage for unmet need - 12.7% reported in the national report [23], given this study computed unmet need based on persons who provided a reason for not seeking outpatient or inpatient care. This means there are persons who did not provide any reason why they did not seek care when needed. Additionally, the national report highlights high cost among the top three most important reasons for not seeking care at 21.4% [23]. Our results emphasize cost as the major reason for not seeking healthcare at 38.6%. This is in accordance with other studies in Kenya, and elsewhere that highlight cost as an important and most frequent reason for unmet need for care [25, 44–46].

The findings slightly differ from those of the national report because we define cost-related unmet need as a combination of two reasons “lacked money” and “high costs of care”, whereas the national report focused on only the high cost of care. The lack of money could infer to other indirect costs of the service such as, transport costs like in rural areas where travel distances to the health facilities are longer [3, 47].

Overall, of those who needed outpatient or inpatient services, 3.2% had to forgo the healthcare services due to cost-related issues (lack of money and high costs). We observe a higher cost-related unmet need for inpatient services relative to outpatient services. This could be because inpatient care is reported to be unaffordable to poor households because it is more costly than outpatient services [48].

Cost-related unmet need varies across the regions/counties ranging from 0.56 to 9.8%. However, 43% of the 44 counties (*n* = 19) do report cost-related unmet need higher than the average in the whole country. This is consistent with other studies in Kenya that have highlighted that health outcomes remain heterogeneous at the county level, although with convergence across counties overtime [49]. This means that cost–related unmet need might impose inequities in access to healthcare between counties. This could be also due to variation in socio-economic status across the counties. Our analysis also shows a positive association between the poverty rates of the counties, and cost-related unmet need for healthcare services. Consistent with other studies, results indicate that counties with high poverty rates had high cost-related unmet need, signifying that health costs are much more a burden to the poor [50].

We assessed the predisposing, need and enabling factors that are associated with cost-related unmet need for care through both a general logistic regression, and multilevel model to account for country level variations. Although there is minimal difference in the estimates of the determinants between the two models, the test for goodness of fit indicate that the multilevel model is superior (better fit) relative to the logistic regression model.

The results indicate that differences between counties accounted for 35.9% of the total variance in inpatient services unmet need, compared to 9.1% in outpatient services unmet need. Furthermore, a wide variation is reported in per capita spending on inpatient care by county [23]. This corroborates previous evidence that there exist inequities within the counties, and that Kenyans living in the same region could have different lifestyles and access to services [51]. Conversely, other studies have highlighted wider socio-economic and geographic inequities for inpatient care than outpatient care in Kenya [52]. However, there are likely to be more similarities among people within the same county in relation to cost-related unmet need for inpatient services relative to outpatient services. Contextual factors at the county level are likely to influence the individual factors on cost-related unmet need. For instance the degree of urbanity at county level, bearing in mind some counties are more urbanised than others and the rural urban disparities in Kenya [53]. This is consistent with studies elsewhere [54], moreover this study also noted a positive significant association between urban residence and cost-related unmet need. This confirms that place(region) does influence health seeking behaviour, like in this case of unmet need due to cost [55, 56].

The results show the positive and negative factors that influence cost-related unmet need controlling for county level variations. Age of household head increased the likelihood of cost-related unmet need for healthcare. This is possibly because older heads are reported to utilize alternative services like self-medication and traditional care [57]. An older age of the head of the household has been reported to increase health care spending [58], resulting in unmet need for care due to high costs. This is in accordance with inequalities in health status that younger people are in general healthier than older people [59]. Furthermore, some studies have shown that households headed by older people are more likely to experience higher health financial burden [14].

Households with an educated head were less likely to forgo care due to cost. Furthermore, lack of formal education is seen as a predictor of poverty given education opens up a range of income–generating opportunities [60]. Other studies have also reported lower levels of education increases the odds of having unmet need [61].

As expected, the need for inpatient services was associated with a higher risk of cost-related unmet need for care relative to outpatient services. This is because inpatient services are required for more severe illnesses [62], hence demanding high costs of care. Moreover, people may have spent more money for consultation prior to needing inpatient services hence depleted their only savings. Compared with other countries in the region (Ghana, Uganda, Zambia), Kenya is reported to have the highest average cost per inpatient bed- day, at about ($41) per day [63], which hinder more people from utilizing the services.

Good self-rated health was associated with a less likelihood of unmet need for healthcare due to cost. This is consistent with other studies that report those with fair, good or very good self-reported health have a lower likelihood of foregone care, which decreases with better health status [64]. People with poor self-rated health are reported to frequent use of medical services [65]. Consistent with other studies, those from insured households had decreased odds of unmet need for care due to costs relative to uninsured households. Social healthcare insurance is reported to significantly reduce foregone care in outpatient and inpatient situations [64]. Elsewhere, financial risk protection through insurance is reported to minimize the prevalence of unmet need due to cost [66].

The larger the household, the less likely they are to forgo care due to cost this is perhaps due to reported pooling of resources among the many members [67] which is common in the African context. Despite the analysis showing more rural households (70.3%) compared to urban households had cost-related unmet need for care, urban households had a positive association with cost-related unmet need. This is corroborated in another study in Kenya that established that forgone care is more heavily concentrated in urban areas [68] especially within the lowest income quintile. Furthermore in Kenya, rural households are reported to have significantly lower OOPs per capita compared to urban households [52]. There is also greater use of expensive health services, and higher costs charged in urban areas relative to rural areas [25]. Additionally, a significant proportion of urban residents (60 to 80%) in Kenya live in informal settlements where a substantial proportion face CHE, and are likely to forgo needed health care because they cannot afford [69]. In many cases, urban households are seen to spend more on OOP for healthcare than rural households, in absolute terms [70]. Consistent with another study in Kenya that foregone care was related to some extent to the available financial resources in a household [71], our findings show a negative association between household wealth and cost-related unmet need. Economic issues are reported to be the primary reason for not seeking healthcare [72]. Elsewhere, studies have shown that unmet needs for health care due to cost are consistently higher among people in low income groups compared with those in high income groups [73, 74]. This implies that wealthy households are less likely to forgo care due to cost, given when prices are high, the poor are more likely than the non-poor to forgo health services [50].

There are limitations to this study that need to be taken into account while interpreting the findings. First, we use the study population as those who reported to have been ill in the 4 weeks preceding the survey and/or needed to be admitted in the past 1 year. There could be differences in the ability of respondents to report illness or interpret illness differently across population groups. For instance, some people only report serious illness and not what they perceive as “less” serious. Second, the outpatient services focus on only the 4 weeks preceding the survey which could fail to capture some segments of the population given the time of fielding the study. This is because some leading causes of illnesses like Malaria are seasonal, that is they are prevalent at particular times of the year and in certain regions. Third, our analysis is based on a subjective unmet need assessment, i.e. the individuals’ self-reported reasons for not seeking care. Previous evidence suggests that perception of health status varies by individual characteristics in that some individuals are better able to estimate their health and their health care needs than others. This study relies on the assumption that the response provided by the respondent was the main reason why care was not sought when needed, and hence could be biased because of the subjective interpretation.

## Conclusions

Controlling for the county level significantly improved the model relative to fitting the general logistic regression. An important note we draw from fitting the multilevel model is the existence of disparities in cost-related unmet need between and within the counties. It is further evident that there exists variation across the regions that could be substantial in explaining the effect of the individual factors on cost-related unmet need. This implies that regional differences are important in explaining health access due to cost, furthermore the counties have varying socio-economic profiles that could predispose the populations to certain health seeking behaviour. The fact that inpatient services was associated with a higher likelihood of cost-related unmet need, highlights the nuances in measuring OOP payments. This shows that OOP for inpatient services as a fraction of household budget could be underestimated as many people may not seek the services due to cost barriers.

The study underscores that affordability is a serious issue in access to healthcare services. The findings suggest that there are multiple factors that drive unmet need of healthcare services due to cost. This requires a multifaceted approach to addressing inequities in unmet need for care, especially among the most vulnerable and marginalised populations. For instance, under the 2010 constitution Kenya setup an ‘equalization fund’ to address unequal distribution of resources with counties as the unit of distribution. The equalization fund employs a county development index to allocate the funds using four parameters namely; poverty, infrastructure, health and education, to measure need. However, the indicators used to measure access to health are specific to certain services that is maternal health, immunization, and sanitation. This study emphasizes the need to reconsider access to health indicators in computing the county development index, to include overall access to healthcare services thus ensure ‘fair’ distribution of resources and address access barriers across the counties.

Health insurance is seen to lower the likelihood of cost-related unmet need, stressing that providing health insurance to the uninsured is key to enabling access. Scaling up of the social health insurance scheme would fundamentally require further segmentation of the population for better targeting, and to ensure it is tailored to match the needs of the various population segments. The current social health insurance scheme is based on a voluntary mechanism which disadvantages the non-employed and poorest given they cannot afford the ‘blanket’ $60 annual premiums. This calls for the need to consider significant improvements to the current insurance package by possibly subsidising premiums or giving exemptions for the most vulnerable and the poorest to improve equity in access.

## Supplementary information

**Additional file 1.** Distribution of cost-related unmet need and other reasons for unmet need for healthcare across the counties (regions).

**Additional file 2.** Distribution of the individuals with cost-related unmet need by socio-economic characteristics.

**Additional file 3.** Multilevel and logistic regression models.

## Data Availability

The datasets generated and/or analysed during the current study are available in the Kenya National Data Archive (KeNADA) repository http://statistics.knbs.or.ke/nada/index.php/home, upon request.

## Abbreviations

OOP: Out of Pocket
CHE: Catastrophic Health Expenditure
UHC: Universal Health Coverage
KHHEUS: Kenya household health expenditure and utilization Survey
ICC: Intraclass Correlation Coefficient
AIC: Alkaike’s information Criterion
BIC: Bayesian Information Criterion
