# Supplementary material for: Cost–related unmet need for healthcare services in Kenya
Source: BMC Health Serv Res. 2020 Apr 17;20:322. doi: 10.1186/s12913-020-05189-3 (PMC7164162; doi:10.1186/s12913-020-05189-3)
Supplement: Supplementary file 1 — Additional file 1. Distribution of cost-related unmet need and other reasons for unmet need for healthcare across the counties (regions). [file 12913_2020_5189_MOESM1_ESM.docx]

**Distribution of cost-related unmet need and other reasons for unmet need for healthcare across the counties(regions)**

|  | **A** | **B** | **D** | **E** | **F** | **G** |
| --- | --- | --- | --- | --- | --- | --- |
| **County** | **Cost-related unmet need (%) D/G** | **Other reasons for unmet need (%) E/G** | **Cost-related unmet need (N)** | **Other reasons for unmet need (N)** | **Total Unmet need (N)** | **Total ill/needed admission (N)** |
| Baringo | 0.76 | 3.15 | 6 | 25 | 31 | 794 |
| Bomet | 0.84 | 6.53 | 8 | 62 | 70 | 950 |
| Bungoma | 4.09 | 4.01 | 48 | 47 | 95 | 1,173 |
| Busia | 6.9 | 4.63 | 82 | 55 | 137 | 1,189 |
| Elgeyo Marakwet | 1.36 | 4.07 | 8 | 24 | 32 | 590 |
| Embu | 3.95 | 5.98 | 37 | 56 | 93 | 936 |
| Homabay | 4.15 | 2.15 | 54 | 28 | 82 | 1,300 |
| Isiolo | 4.92 | 1.97 | 35 | 14 | 49 | 712 |
| Kajiado | 0.56 | 3.93 | 4 | 28 | 32 | 712 |
| Kakamega | 3.78 | 1.79 | 57 | 27 | 84 | 1,506 |
| Kericho | 3.17 | 17.92 | 37 | 209 | 246 | 1,166 |
| Kiambu | 1.63 | 3.54 | 12 | 26 | 38 | 734 |
| Kilifi | 2 | 4.95 | 21 | 52 | 73 | 1,050 |
| Kirinyaga | 1.11 | 3.02 | 11 | 30 | 41 | 994 |
| Kisii | 4.26 | 5.60 | 35 | 46 | 81 | 822 |
| Kisumu | 1.6 | 0.80 | 16 | 8 | 24 | 1,002 |
| Kitui | 5.89 | 6.22 | 71 | 75 | 146 | 1,206 |
| Kwale | 3.92 | 2.72 | 39 | 27 | 66 | 994 |
| Laikipia | 3.01 | 3.76 | 20 | 25 | 45 | 665 |
| Lamu | 1.93 | 7.72 | 19 | 76 | 95 | 984 |
| Machakos | 3.27 | 4.73 | 47 | 68 | 115 | 1,438 |
| Makueni | 3.53 | 5.23 | 31 | 46 | 77 | 879 |
| Marsabit | 6.47 | 7.58 | 35 | 41 | 76 | 541 |
| Meru | 1.75 | 4.29 | 18 | 44 | 62 | 1,026 |
| Migori | 2.12 | 0.90 | 33 | 14 | 47 | 1,557 |
| Mombasa | 4.03 | 4.17 | 28 | 29 | 57 | 695 |
| Murang'a | 1.91 | 1.91 | 19 | 19 | 38 | 994 |
| Nairobi | 1.59 | 4.34 | 15 | 41 | 56 | 944 |
| Nakuru | 3.7 | 14.95 | 23 | 93 | 116 | 622 |
| Nandi | 4.28 | 0.86 | 35 | 7 | 42 | 818 |
| Narok | 3.03 | 5.52 | 22 | 40 | 62 | 725 |
| Nyamira | 1.95 | 1.39 | 21 | 15 | 36 | 1,078 |
| Nyandarua | 0.94 | 3.24 | 9 | 31 | 40 | 957 |
| Nyeri | 1.16 | 4.25 | 9 | 33 | 42 | 777 |
| Samburu | 4 | 26.71 | 34 | 227 | 261 | 850 |
| Siaya | 1.66 | 3.39 | 20 | 41 | 61 | 1,208 |
| Taita-Taveta | 0.9 | 4.86 | 7 | 38 | 45 | 782 |
| Tanariver | 9.8 | 7.16 | 100 | 73 | 173 | 1,020 |
| Tharaka Nithi | 1.98 | 2.64 | 21 | 28 | 49 | 1,061 |
| Trans Nzoia | 4.21 | 15.23 | 45 | 163 | 208 | 1,070 |
| Turkana | 9.67 | 1.56 | 62 | 10 | 72 | 641 |
| Uasin Gishu | 4.26 | 4.49 | 36 | 38 | 74 | 846 |
| Vihiga | 2.12 | 1.02 | 25 | 12 | 37 | 1,180 |
| West Pokot | 3.15 | 4.86 | 22 | 34 | 56 | 699 |
| **Total** |  |  | **1,337** | **2,125** | **3,462** | **41,887** |
