# Supplementary material for: Cost–related unmet need for healthcare services in Kenya
Source: BMC Health Serv Res. 2020 Apr 17;20:322. doi: 10.1186/s12913-020-05189-3 (PMC7164162; doi:10.1186/s12913-020-05189-3)
Supplement: Supplementary file 2 — Additional file 2. Distribution of the individuals with cost-related unmet need by socio-economic characteristics. [file 12913_2020_5189_MOESM2_ESM.docx]

**Distribution of the individuals with cost-related unmet need by socio-economic characteristics**

| **Variables** | **Categories** | **Outpatient (%)** | **Inpatient (%)** | **Total (%)** | **Sample**  **N (1,337)** |
| --- | --- | --- | --- | --- | --- |
| **Predisposing factors** |  |  |  |  |  |
| Age group of HH | Below 25 Years | 2.78 | 2.73 | 2.77 | 37 |
|  | 25-40 Years | 30.99 | 41.41 | 32.98 | 441 |
|  | 40 + Years | 66.23 | 55.86 | 64.25 | 859 |
| HH head education level | None | 1.85 | 1.98 | 1.88 | 25 |
|  | Primary education | 81.37 | 80 | 81.08 | 1,080 |
|  | Secondary education | 14.27 | 13.04 | 14.04 | 187 |
|  | Tertiary | 2.5 | 5.14 | 3 | 40 |
| Gender of HH | Male | 68.36 | 67.19 | 68.14 | 911 |
|  | Female | 31.64 | 32.81 | 31.86 | 426 |
| Employment status of HH head | Unemployed | 16.93 | 19.92 | 17.5 | 234 |
|  | Employed | 83.07 | 80 | 82.5 | 1,103 |
| **Need factors** |  |  |  |  |  |
| Type of service | Outpatient | NA | NA | 80.85 | 1081 |
|  | Inpatient | NA | NA | 19.15 | 256 |
| Self-rated health | Poor | 14.26 | 29.69 | 17.22 | 230 |
|  | Satisfactory | 27.41 | 22.66 | 26.5 | 354 |
|  | Good | 58.33 | 47.66 | 56.29 | 752 |
| Chronic illness | No chronic illness | 75.95 | 64.84 | 73.8 | 987 |
|  | Chronic illness | 24.05 | 35.16 | 26.18 | 350 |
| **Enabling factors** |  |  |  |  |  |
| Household size | 1-3 Small Size | 26.85 | 22.66 | 26.05 | 348 |
|  | 4-6 Medium Size | 43.24 | 38.28 | 42.29 | 565 |
|  | 7+ Large Size | 29.91 | 39.06 | 31.66 | 423 |
| Insurance status | Not insured | 93.43 | 90 | 92.74 | 1,240 |
|  | Insured | 6.57 | 10.16 | 7.26 | 97 |
| Residence | Rural | 70.21 | 70.7 | 70.31 | 940 |
|  | Urban | 29.79 | 29.3 | 29.69 | 397 |
| Wealth quintile | Poorest | 38.61 | 35.94 | 38.1 | 509 |
|  | Second | 25.93 | 24.61 | 25.67 | 343 |
|  | Middle | 19.63 | 17.97 | 19.31 | 258 |
|  | Fourth | 10.74 | 17.97 | 12.13 | 162 |
|  | Richest | 5.09 | 3.52 | 4.79 | 64 |

*HH Household; NA Not applicable*
