# Supplementary material for: Cost–related unmet need for healthcare services in Kenya
Source: BMC Health Serv Res. 2020 Apr 17;20:322. doi: 10.1186/s12913-020-05189-3 (PMC7164162; doi:10.1186/s12913-020-05189-3)
Supplement: Supplementary file 3 — Additional file 3. Multilevel and logistic regression models. [file 12913_2020_5189_MOESM3_ESM.docx]

**Multilevel and logistic regression models**

| **Factors** | **Categories** | **Multilevel Model** | | **Logistic regression** | |
| --- | --- | --- | --- | --- | --- |
|  |  | **Odds Ratio** | **[95% CI]** | **Odds Ratio** | **[95%CI]** |
| **Predisposing factors** |  |  |  |  |  |
| *Gender of the HH head* | Male [RC] |  |  |  |  |
|  | Female | 1.019 | (0.897 -1.157) | 0.968 | (0.854-1.096) |
| *Age group of the HH head* | Below 25years [RC] |  |  |  |  |
|  | 25-40 years | 1.333 | (0.936-1.898) | 1.198 | (0.843-1.700) |
|  | 40+ years | 1.793*** | (1.264-2.545) | 1.559 ** | (1.103-2.204) |
| *Education level of HH head* | None [RC] |  |  |  |  |
|  | Primary | 0.956 | (0.624-1.465) | 0.736 | (0.487-1.112) |
|  | Secondary | 0.669 | (0.426-1.052) | 0.509 ** | (0.329-0.788) |
|  | Tertiary | 0.534* | (0.310-0.921) | 0.423*** | (0.249-0.718) |
| *Employment status of HH head* | Not employed [RC] |  |  |  |  |
|  | Employed | 0.932 | (0.786-1.103) | 0.725 *** | (0.623-0.843) |
| **Need factors** |  |  |  |  |  |
| *Type of service* | Outpatient [RC] |  |  |  |  |
|  | Inpatient | 1.950*** | (1.683-2.260) | 1.961 *** | (1.697-2.265) |
| *Self-rated health* | Poor [RC] |  |  |  |  |
|  | Satisfactory | 0.942 | (0.786-1.129) | 0.98 | (0.821-1.170) |
|  | Good | 0.567*** | (0.477-0.673) | 0.598 *** | (0.505-0.707) |
| *Chronic Illness* | No Illness [RC] |  |  |  |  |
|  | Chronic illness | 1.067 | (0.924-1.231) | 1.077 | (0.935-1.241) |
| **Enabling factors** |  |  |  |  |  |
| *Insurance status* | Not insured [RC] |  |  |  |  |
|  | Insured | 0.505*** | (0.401-0.636) | 0.463 *** | (0.369-0.580) |
| *Household size* | 1-3 Small [RC] |  |  |  |  |
|  | 4-6 Medium Size | 0.693*** | (0.600-0.802) | 0.732 *** | (0.635-0.845) |
|  | 7+ Large Size | 0.717*** | (0.611-0.842) | 0.816 ** | (0.698-0.953) |
| *Residence* | Rural [RC] |  |  |  |  |
|  | Urban | 1.259*** | (1.094-1.449) | 1.274 *** | (1.116-1.454) |
| *Wealth Index* | Poorest [RC] |  |  |  |  |
|  | Second quintile | 0.845* | (0.724-0.987) | 0.735 *** | (0.638-0.848) |
|  | Middle quintile | 0.632*** | (0.532-0.751) | 0.585 *** | (0.499-0.686) |
|  | Fourth quintile | 0.470*** | (0.380-0.579) | 0.440 *** | (0.361-0.535) |
|  | Richest quintile | 0.314*** | (0.226-0.436) | 0.324 *** | (0.240-0.439) |
|  | _cons | 0.055*** | (0.030-0.103) | 0.112 *** | (0.064-0.198) |
| Random effects | /lnsig2u [County variance] | -1.074 | (-1.573- –0.574) |  |  |
|  | sigma_u [Residual variance] | 0.585 [0.074] | (0.455-0.750) |  |  |
|  | Rho [Intraclass correlation (ICC)] | 0.094 [0.022] | (0.059-0.146) |  |  |
|  | Log likelihood | -5426.35 |  | -5554.9141 |  |
|  | Wald chi2(19) | 515.03 |  | 668.35 |  |
|  | Number of observations | 41,646 |  | 41,646 |  |
|  | Number of groups | 44 |  |  |  |

*Log likelihood ratio test: logistic Vs multilevel regression: chi2(2) =257.137, Prob <0.001*

*P < 0.05*, 0.01** and 0.001***, Ref Reference, HH Household*
